# 640 Unemployed Burn Survivors Report Significantly Greater Usage of Antidepressants and Anxiolytics

**DOI:** 10.1093/jbcr/iraf019.269

**Published:** 2025-04-01

**Authors:** Deborah Choe, Ayumi Saito, Andrew Humbert, Kimberly Roaten, Karin Blen, Jeffrey Schneider, Juan Herrera-Escobar, Haig Yenikomshian

**Affiliations:** University of California Keck School of Medicine; University of Washington; University of Washington; University of Texas Southwestern; Los Angeles General Medical Center; Spaulding Rehabilitation Hospital, Harvard Medical School; Brigham and Women’s Hospital, Harvard Medical School; University of California Keck School of Medicine

## Abstract

**Introduction:**

Burn injuries can result in significant physical pain as well as psychosocial distress which can last months after injury. Oftentimes, opioids, antidepressants, and anxiolytic medications are used to help with these issues. At the same time, long-term use of these medications can result in side effects and complications. Risk factors for long-term use are poorly understood. One potential risk factor that has not been well studied is preinjury employment status. Unemployment may be associated with physical and psychosocial distress, but connection between employment status and medication usage for pain, anxiety, and depression among burn survivors is unknown. We aimed to compare long-term, self-reported use of pain, depression, and anxiety medication between adults who were unemployed and employed before their burn injury.

**Methods:**

Adult burn survivors participating in a national longitudinal, multicenter patient-reported outcomes database between 2015-2023 were included. Participants were divided into two groups: those who were employed at the time of injury and those who were unemployed. Survey responses reporting pain, depression, and anxiety medication usage at 6-, 12-, and 24-months post-burn injury were analyzed. Associations between pre-burn employment status and long-term outcome scores were analyzed using mixed-effects models while adjusting for sex, age, TBSA burn size, presence of the COVID-19 pandemic, and time after injury.

**Results:**

A total of 814 participants were included, of which 635 were employed and 179 were unemployed pre-burn injury. Compared to those employed pre-burn injury, unemployed burn participants reported significantly higher rates of using depression (13%, p< 0.01) and anxiety medications (14% higher, p< 0.01), after adjusting for sex, age, TBSA burn size, presence of the COVID-19 pandemic, and time after injury. However, there were no significant differences in usage of pain medication (p=0.58) between groups.

**Conclusions:**

These findings indicate that unemployed burn survivors use depression and anxiety medications more frequently long-term. Our study suggests that unemployment status should be considered when discussing long-term recovery and psychosocial outcomes among adults living with burn injuries. This may be necessary due to the financial constraints of not working as well as the feelings of hopelessness and isolation associated with unemployment. Unemployed burn survivors may benefit from proactive screening to detect emotional distress and early, non-pharmacological interventions such as avocational activities or psychotherapy to treat symptoms. Vocational counseling may also be effective for identifying opportunities to return to work.

**Applicability of Research to Practice:**

This study may be considered when recommending psychotherapy and/or community groups for adults living with burn injuries.

**Funding for the Study:**

The contents of this abstract were developed under a grant from the National Institute on Disability, Independent Living, and Rehabilitation Research. NIDILRR is a Center within the Administration for Community Living (ACL), Department of Health and Human Services (HHS). The contents of this abstract do not necessarily represent the policy of NIDILRR, ACL, or HHS, and you should not assume endorsement by the Federal Government.

